# The Mutational Spectrum of Lynch Syndrome in Cyprus

**DOI:** 10.1371/journal.pone.0105501

**Published:** 2014-08-18

**Authors:** Maria A. Loizidou, Ioanna Neophytou, Demetris Papamichael, Panteleimon Kountourakis, Vassilios Vassiliou, Yiola Marcou, Eleni Kakouri, Georgios Ioannidis, Chrystalla Philippou, Elena Spanou, George A. Tanteles, Violetta Anastasiadou, Andreas Hadjisavvas, Kyriacos Kyriacou

**Affiliations:** 1 Department of Electron Microscopy/Molecular Pathology, The Cyprus Institute of Neurology and Genetics, Nicosia, Cyprus; 2 Departments of Medical and Radiation Oncology, Bank of Cyprus Oncology Center, Nicosia, Cyprus; 3 Department of Oncology, Nicosia General Hospital, Nicosia, Cyprus; 4 Department of Oncology, Limassol General Hospital, Limassol, Cyprus; 5 Clinical Genetics Department, Makarios III Hospital and The Cyprus Institute of Neurology and Genetics, Nicosia, Cyprus; Deutsches Krebsforschungszentrum, Germany

## Abstract

Lynch syndrome is the most common form of hereditary colorectal cancer and is caused by germline mutations in the mismatch repair (MMR) genes *MLH1*, *MSH2*, *MSH6* and *PMS2*. Mutation carriers have an increased lifetime risk of developing colorectal cancer as well as other extracolonic tumours. The aim of the current study was to evaluate the frequency and distribution of mutations in the *MLH1*, *MSH2* and *MSH6* genes within a cohort of Cypriot families that fulfilled the revised Bethesda guidelines. The study cohort included 77 patients who fulfilled at least one of the revised Bethesda guidelines. Mutational analysis revealed the presence of 4 pathogenic mutations, 3 in the *MLH1* gene and 1 in the *MSH2* gene, in 5 unrelated individuals. It is noted that out of the 4 pathogenic mutations detected, one is novel (c.1610delG in exon 14 of the *MLH1*) and has been detected for the first time in the Cypriot population. Overall, the pathogenic mutation detection rate in our patient cohort was 7%. This percentage is relatively low but could be explained by the fact that the sole criterion for genetic screening was compliance to the revised Bethesda guidelines. Larger numbers of Lynch syndrome families and screening of the two additional predisposition genes, *PMS2* and *EPCAM*, are needed in order to decipher the full spectrum of mutations associated with Lynch syndrome predisposition in Cyprus.

## Introduction

Colorectal cancer (CRC) is the second most commonly diagnosed cancer in Europe [1]. It is estimated that up to 6% of all CRCs can be attributed to a known predisposition syndrome [2]. Lynch syndrome (LS), also referred to as Hereditary Non-Polyposis Colorectal Cancer (HNPCC) syndrome, is the most common form of hereditary CRC [3]. It is caused by germline mutations in the mismatch repair (MMR) genes *MLH1*, *MSH2*, *MSH6* and *PMS2* and is inherited in an autosomal dominant manner. Recently, the *EPCAM* gene, which is located upstream of the *MSH2* gene, has also been implicated in LS [4]. It is estimated that LS, accounts for about 2–4% of the total CRC burden [2], [5] and that the prevalence of the MMR gene mutations in the general population is 1 in 300–500 individuals [6].

Individuals with a germline mutation in one of the LS predisposition genes are at an increased risk of developing CRC as well as other extracolonic tumours including endometrial, ovarian, gastric, urothelial, pancreatic and sebaceous gland tumours [7]. Since the discovery of the LS predisposition genes, a number of studies have attempted to quantify the cancer risk associated with this syndrome. The first published studies have reported lifetime risks of colorectal and endometrial cancer reaching 80% and 60% respectively [8], [9]. However, more recent studies which have corrected for ascertainment bias, estimated a lifetime risk for CRC ranging between 22% and 66% and for endometrial cancer ranging between 32 and 45% [10], [11], [12]. Individuals found to carry a pathogenic mutation in one of the LS predisposition genes can benefit from targeted cancer surveillance protocols and risk-reducing surgical options which can help reduce mortality by about 65% [13].

In order to identify individuals at-risk for LS, clinical criteria, namely the Amsterdam criteria [14] and the Bethesda guidelines[15], have been developed and subsequently revised [16], [17] to increase their sensitivity. The diagnostic value of the Amsterdam criteria has been questioned since it has been shown that 40% of families with known mutations do not meet the Amsterdam criteria and 50% of those who fulfill the criteria do not have a detectable gene defect [18]. Furthermore, it has been reported that up to 28% of mutation carriers can be missed when testing is limited to individuals, who strictly meet the Bethesda guidelines [7], [19].

According to the revised Bethesda guidelines patients who fulfill the following criteria should be recommended to undergo genetic testing: a) those diagnosed with CRC before the age of 50 years b) those who had synchronous or metachronous colorectal or other LS-associated tumours, regardless of age c) those diagnosed with CRC under the age of 60 years whose tumour biopsy had microsatellite instability (MSI-H) c) those who had one or more first-degree relatives with CRC or other LS-related tumour, with one of the cancers being diagnosed by the age of 50 years d) those who had CRC with two or more first- or second-degree relatives with CRC or other LS-related tumours, regardless of age [17]. The aim of this study was to evaluate the frequency and distribution of mutations in the *MLH1*, *MSH2* and *MSH6* genes within a cohort of Cypriot families which fulfilled the revised Bethesda guidelines and were hence eligible for genetic testing.

## Materials and Methods

### Ethics statement

Written informed consent was obtained from all subjects. The study was approved from the Cyprus Institute of Neurology and Genetics.

### Patient recruitment

The study cohort included 77 patients who fulfilled at least one of the revised Bethesda guidelines [17]. Patients were referred by their treating oncologists to the Cancer Genetics Clinic of the Cyprus Institute of Neurology and Genetics where they were offered genetic counseling. Genetic counseling for cancer predisposition involves a comprehensive personal risk assessment based on medical and family history, a detailed discussion of options including genetic testing and preventative screening and support throughout the decision making process. When indicated, genetic testing was offered. All patients signed an informed consent form prior to molecular genetic testing. Results were communicated to the patients/families through a second genetic counseling appointment.

### Mutation detection

Genomic DNA was isolated from peripheral blood lymphocytes using standard extraction protocols. Polymerase chain reaction (PCR) was used to amplify the entire coding sequence and intron–exon junctions of the *MLH1*, *MSH2* and *MSH6* genes. Following PCR amplification, direct sequencing was carried out using the ABI PRISM di-Deoxy Terminator Cycle sequencing kit v 3.1 on an ABI 9700 thermal cycler and an ABI 3130XL Genetic Analyzer (Applied Biosystems, Foster City, CA, USA). The PCR products were sequenced using the same primers as the ones used for PCR amplification. When a mutation was identified, a new PCR product using a second DNA sample was sequenced so as to confirm the result.

In order to detect large genomic rearrangements in the *MLH1* and *MSH2* genes, multiplex ligation-dependent probe amplification (MLPA, MRC Holland, Amsterdam, The Netherlands) using the P003 kit was carried out following the manufacturer's instructions. Fragment analysis was carried out on an ABI 3130XL Genetic Analyzer using LIZ-500 as a size standard.

## Results

Seventy seven patients were screened for the presence of germline mutations in the *MLH1*, *MSH2* and *MSH6* genes. When a pathogenic mutation was identified, other at-risk relatives were subsequently tested for the presence of the same mutation. In total, 144 samples were subjected to genetic testing.

Mutational analysis of the three main LS predisposition genes revealed the presence of 4 pathogenic mutations (3 in the *MLH1* gene and 1 in the *MSH2* gene) in 5 unrelated individuals who fulfilled the revised Bethesda guidelines. The deleterious mutation details along with the patients' clinical and family history data are summarized in Table 1.

**Table 1:** Summary of the pathogenic mutations detected and family history of the probands.

#	Diagnosis	Gene	Exon	DNA change+	Protein	Family History++
1	CRC 40 & 75	*MLH1*	9i	c.790+1G>A	p.Glu227_Ser295del	CRC 57 & EC (daughter), 5 CRC cases in first degree relatives
2	CRC 38	*MLH1*	13	c.1410-?_1558+?del	p.Arg470Serfs*8	6 cases of CRC in first degree relatives
3	CRC 54	*MLH1*	13	c.1410-?_1558+?del	p.Arg470Serfs*8	RC 49 (brother)
4	CRC 33	*MLH1*	14	c.1610delG	p.Gln537Hisfs*54	BrC 17 (brother), BrC 60 (maternal aunt)
5	CRC 32	*MSH2*	12	c.1968C>G	p.Tyr656*	EC (sister), CRC (mother), CRC (maternal grandmother)

+ Mutation nomenclature according to NM_000249.3 (*MLH1*), NM_000251.2 (*MSH2*) and NM_000179.2 (*MSH6*). For the nomenclature of mutations nucleotide 1 is the A of the ATG-translation initiation codon.

++ BrC – brain cancer.

CRC – colorectal cancer.

EC – endometrial cancer.

RC – renal cancer.

The range of deleterious mutations detected in our patient cohort includes a large deletion of exon 13 of the *MLH1* gene (c.1410-?_1558+?del) detected in two unrelated individuals, a frameshift mutation in exon 14 of the *MLH1* gene (c.1610delG), a splice site mutation in intron 9 of the *MLH1* gene (c.790+1G>A) and a nonsense mutation in exon 12 of the *MSH2* gene (c.1968C>G). The frameshift mutation in the *MLH1* gene is novel whereas the other 3 mutations have been reported before several times in the InSiGHT database [20].

In addition, 13 missense mutations have been detected in our patient cohort; three in the *MLH1* gene, five in the *MSH2* and five in the *MSH6* gene (Table 2). Out of the three missense mutations identified in the *MLH1* gene, one (c.1852_1853delinsGC) has been classified as a Class 1 variant (not pathogenic) by the international panel of researchers and clinicians of the InSiGHT consortium [21], whereas the other two (c.1360G>C and c.1652A>C) are of uncertain clinical significance (Class 3). Accordingly, out of the five missense mutations identified in the *MSH2* gene, one (c.965G>A) is not pathogenic according to the InSiGHT consortium (Class 1), whereas three (c.182A>C, c.435T>G and c.1667T>C) are of uncertain clinical significance (Class 3) and the remaining one (c.992A>C) has not been reported previously. In addition, five missense mutations have been detected in the *MSH6* gene, a class 1 variant (c.2633T>C), two class 3 variants (c.1729C>T and c.2092C>G) and two novel variants (c.1069G>A and c.1539C>G).

**Table 2:** Summary of the missense mutations identified.

Gene	Exon	DNA change+	Protein	InSiGHT Class++	Diagnosis+++
***MLH1***					
	12	c.1360G>C	p.Gly454Arg	Class 3	CRC 65 & CRC 43
	14	c.1652A>C	p.Asn551Thr	Class 3	OC 38 & 43, CRC 45, BC 48
	16	c.1852_1853delinsGC	p.Lys618Ala	Class 1	CRC 47
***MSH2***					
	1	c.182A>C	p.Gln61Pro	Class 3	OC 44, CRC 50
	3	c.435T>G	p.Ile145Met	Class 3	CRC 51
	6	c.965G>A	p.Gly322Asp	Class 1	OC 41
	6	c.992A>C	p.Asn331Ser	Novel	GC 57, OC 59
	11	c.1667T>C	p.Leu556Ser	Class 3	CRC 35
***MSH6***					
	4	c.1069G>A	p.Asp357Asn	Novel	OC 29
	4	c.1539C>G	p.Ile513Met	Novel	CRC 49
	4	c.1729C>T	p.Arg577Cys	Class 3	CRC 49
	4	c.2092C>G	p.Gln698Glu	Class 3	CRC 65
	4	c.2633T>C	p.Val878Ala	Class 1	CRC 69

+ Mutation nomenclature according to NM_000249.3 (*MLH1*), NM_000251.2 (*MSH2*) and NM_000179.2 (*MSH6*). For the nomenclature of mutations nucleotide 1 is the A of the ATG-translation initiation codon.

++ Class 1: not pathogenic.

Class 3: uncertain.

Class 5: pathogenic.

+++ BC – breast cancer.

CRC – colorectal cancer.

GC – gastric cancer.

OC – ovarian cancer.

The pathogenicity of the three novel variants identified in the *MSH2* and *MSH6* genes has been assessed using Align-GVGD online software [22]. Align-GVGD is a web-based program that combines the biophysical characteristics of amino acids with protein multiple sequence alignments to predict the pathogenicity of missense substitutions in genes of interest [21]. All three variants were predicted not to be pathogenic (Class C0).

## Discussion

Lynch syndrome is the most common cause of hereditary CRC caused by defects in one of the MMR genes [3]. Mutation carriers have an increased lifetime risk for developing colorectal and endometrial cancers as well as a spectrum of other tumours, at an early age of diagnosis. Inactivating *MLH1* and *MSH2* mutations account for the majority of LS cases (∼90%), whereas the remaining 10% of the cases are mainly due to mutations in the *MSH6* and *PMS2* genes [23].

The mutational spectrum of Lynch syndrome predisposition genes varies across different populations. It is also well-established that specific founder mutations prevail in certain ethnic groups [24], [25], [26], [27]. In an attempt to assess the frequency and distribution of *MLH1*, *MSH2* and *MSH6* mutations in Cyprus, a cohort of Cypriot patients which fulfilled the revised Bethesda guidelines were subjected to genetic testing.

A total of four pathogenic mutations, three point mutations and a large genomic deletion have been detected in five out of the seventy seven probands tested. It is noted that three out of the five patients who carried a pathogenic mutation, also fulfilled the revised Amsterdam criteria. Three out of the four pathogenic mutations identified (c.790+1G>A, c.1410-?_1558+?del and c.1610delG) were in the *MLH1* gene whereas the other one (c.1968C>G) was in the *MSH2* gene.

The *MLH1* exon 13 deletion (c.1410-?_1558+?del) has been detected in 35 individuals from two unrelated families. This large deletion has been reported previously in 14 patients from various countries (InSiGHT mutation database, http://www.insight-group.org). In the first affected family, 7 patients with CRC (mean age of diagnosis 42 years) as well as 18 healthy individuals (mean age 32 years) were found to be heterozygous for the *MLH1* exon 13 deletion. Likewise, in the second affected family, two cancer patients (the proband who was diagnosed with CRC at the age of 54 years and his brother who was diagnosed with renal cancer at the age of 49) and 8 healthy individuals (mean age 32 years) were heterozygous for the large deletion encompassing exon 13 of the *MLH1* gene. The identification of Cypriot individuals at risk for colorectal and endometrial cancer at a relatively young age (well below the average age of diagnosis at Lynch syndrome) poses substantial benefits in terms of reduced morbidity and mortality. The long-term effectiveness of surveillance of mutation carriers by colonoscopies with polypectomies and endometrial biopsies with transvaginal ultrasonography is well documented [28]. With the increased availability of screening methods for early detection of premalignant adenomas the healthy carriers from the 2 LS affected families will greatly benefit from a close surveillance program.

The c.790+1G>A intronic substitution leads to an in-frame deletion of exons 9 and 10 of the *MLH1* gene [29]. This mutation has been reported before 34 times in the InSiGHT mutation database and has been detected in a Cypriot family with multiple cases of colorectal and endometrial cancer across four successive generations.

The c.1610delG deletion in exon 14 of the *MLH1* gene has not been reported before. This frameshift mutation introduces a premature stop codon at amino acid 590 of the *MLH1* gene. It was detected in an early onset CRC patient who was diagnosed at the age of 33 years. Interestingly, the proband had no family history of colorectal or endometrial cancers but his deceased brother and a maternal aunt were diagnosed with brain cancer at the ages of 17 and 60 years respectively. The constellation of colorectal cancer and brain tumours, in particular glioblastomas, are associated with Turcot syndrome, a rare molecularly heterogeneous syndrome caused by mutations in the *MLH1* and *PMS2* genes [30].

Only one pathogenic mutation in the *MSH2* gene (c.1968C>G) has been detected in our patient cohort. This is a nonsense mutation which leads to a premature stop codon at position 656 of the *MSH2* gene. It has been reported 8 times before in patients of German, Polish and Australian ancestry (InSiGHT database). The proband of this family was diagnosed with early onset CRC at the age of 32 years and had a family history of colorectal and endometrial cancers across three successive generations.

Thirteen Cypriot patients were found to harbor a missense mutation in either of the *MLH1*, *MSH2* and *MSH6* genes. Missense mutations, pose significant difficulties for management due to their unknown clinical impact [31]. The InSiGHT consortium in an attempt to address this problem have successfully undertaken the task of the classification of hereditary sequence variants identified in LS related genes. They have developed a five-tiered system of classification ranging from class 1 (not pathogenic) to class 5 (pathogenic) [21]. According to this classification, 3 of the missense mutations detected in our patient cohort (one in each gene) are not pathogenic. In addition, *in silico* analysis using Align-GVGD software predicted that the 3 novel mutations detected in the *MSH2* and *MSH6* genes are also not pathogenic. The remaining 7 missense mutations detected, were classified as class 3 variants (variants of unknown clinical significance). It is estimated that around 50% of the *MLH1* and *MSH2* missense variants may be classified as pathogenic after further investigation [21]. Further investigation is warranted by incorporating bioinformatic predictions, segregation information and pathology data in order to elucidate whether the missense mutations identified in our patient cohort affect protein function. Accurate classification of missense mutations will improve genetic counseling, patients' management and lead to a more targeted presymptomatic surveillance of carriers.

Overall, the pathogenic mutation detection rate in our patient cohort was 7%. This percentage is relatively low but could be explained by the fact that the sole criterion for genetic screening was compliance to the revised Bethesda guidelines. It is noted that out of the seventy two patients for which we failed to identify a pathogenic mutation, twenty nine had a family history of at least two additional colorectal or Lynch-related cancers whereas 33 had a young age of onset (<50 years) but no family history.

The revised Bethesda guidelines are less stringent than the Amsterdam criteria and take into account medical and family history of LS-associated tumours as well as early onset of CRC in a single patient within a family [17]. As it has been demonstrated in a study by the German HNPCC consortium [32], families meeting the revised Bethesda guidelines have a much lower MMR mutation frequency compared to families meeting the revised Amsterdam criteria. There are two possible reasons behind this; firstly, it could be due to the fact that affected family members are not as closely related and secondly it may be attributed to the higher age of diagnosis in this group of patients, which may lead to the inclusion of sporadic cancers [32]. The low mutation detection rate in our patient series could also be explained by the inclusion of early onset colorectal cancer patients without a family history of Lynch related cancers. Stigliano et al. have concluded that early-onset colorectal cancer patients, with left sided colorectal cancer and without family history are “at very low risk” for LS [33].

Mutation analysis for Lynch syndrome predisposition is both costly and time-consuming and should be restricted to those individuals who are more likely to carry a mutation. As neither the Amsterdam nor the Bethesda criteria provide the necessary high sensitivity/specificity in a reliable and consistent manner, it is recommended that two pre-screening tests should be included in the diagnostic algorithm [34]. These tests are performed on paraffin embedded tumour tissues, are based on microsatellite instability (MSI) and immunohistochemistry (IHC), and contribute to the identification of those patients that are more likely to benefit from genetic testing. It has been estimated that the specificity of MSI for detecting individuals with LS is 90.2%, while the specificity of IHC is 88.8% [34]. Concurrent screening for the *BRAF* V600E mutation in tumour DNA from MSI positive cases exhibiting loss of MLH1 and PMS2 expression by IHC, will further assist in the exclusion of non-eligible patients [35]. The development of an efficient screening strategy for LS screening will lead to the identification of further Cypriot patients/families who harbor pathogenic mutations.

## Conclusion

This is the first report presenting molecular genetic data of LS in the Cypriot population. Mutational analysis revealed the presence of 4 pathogenic mutations; 3 in the *MLH1* gene (one of which is novel) and 1 in the *MSH2* gene in 5 unrelated individuals. Despite the relatively small number of families studied, it appears that the *MLH1* gene plays a more important role than the *MSH2* gene in Lynch syndrome predisposition in Cyprus. Larger numbers of LS families and screening of the two additional predisposition genes, *PMS2* and *EPCAM* are needed in order to be able to generalize our conclusions and decipher the full spectrum of mutations associated with LS predisposition in Cyprus.
